# Effect of a Polynucleotide-Hyaluronic Acid Gel on Mandibular Third-Molar Extraction Discomfort

**DOI:** 10.1097/SCS.0000000000012307

**Published:** 2025-12-31

**Authors:** Vincenzo Ronsivalle, Gabriele Cervino, Roberta Giudice, Simona Santonocito, Marco Cicciù

**Affiliations:** * Department of General Surgery and Surgical-Medical Specialties, School of Dentistry, University of Catania, Catania; † Department of Biomedical and Dental Sciences, Morphological and Functional Images, University of Messina, G. Martino Polyclinic, Messina, Italy

**Keywords:** Hyaluronic acid, mandibular third-molar extraction, polynucleotides

## Abstract

**Background:**

Postoperative pain, swelling, and transient trismus commonly follow impacted mandibular third-molar surgery. Hyaluronic acid (HA) can modulate early inflammation and support soft-tissue repair, while polynucleotides (PN) may further enhance fibroblast activity. This study evaluated whether an intrasocket PN–HA gel improves early postoperative outcomes versus standard care.

**Methods:**

Single-center, retrospective split-mouth study in adults undergoing bilateral removal of partially impacted mandibular third molars. One socket received PN–HA gel (Regenfast^©^, 5 mL) before suturing (TG), the contralateral socket received no adjunct (CG). Outcomes: pain (VAS 0–10) at 12 hours and daily for 7 days; maximum interincisal distance on 2, 5, and 7 postoperative days (POD); facial swelling from standardized 3D facial scans at baseline, POD2, POD5, and POD7. Parametric analyses used paired tests and one-way ANOVA with Tukey post hoc; Pearson correlations assessed relations among pain, swelling, and mouth opening.

**Results:**

Eighteen patients completed follow-up without complications. Operative time was comparable between allocations. Pain peaked at approximately 12 hours and was lower on test sides during the early window, with differences diminishing by POD5–7. Swelling increases were smaller on test sides at POD2 and POD5 and converged by POD7. MID recovered faster on test sides at POD2 and POD5, approaching baseline in both groups by POD7. Correlations reflected expected dynamics: higher pain was associated with greater swelling and reduced mouth opening, most pronounced around the inflammatory peak.

**Conclusion:**

Intrasocket PN–HA gel reduced early pain and swelling and hastened mouth-opening recovery (greatest at 48–72 h, converging by 1 week), indicating clinical utility for reducing short-term morbidity after third-molar surgery.

Surgical removal of impacted mandibular third molars is a routine procedure in oral and maxillofacial surgery.^1–5^ Indications for extraction include caries involving the third molar or the distal surface of the adjacent second molar; recurrent pericoronitis in partially erupted teeth; external root resorption of the second molar; associated pathology such as cysts or odontogenic tumors; requirements related to orthodontic or prosthetic treatment; orthognathic surgical planning; and orofacial symptoms, including facial discomfort and headaches.^6–11^ An age of approximately 24 years is commonly cited as the upper threshold for third-molar removal, as evidence suggests a higher risk of lasting neurological injury in patients older than this benchmark.^6,12,13^ Potential complications following mandibular third-molar extraction include alveolitis (alveolar osteitis), infection, hematoma, hemorrhage, injury to the inferior alveolar or lingual nerves, mandibular fractures, soft-tissue trauma, iatrogenic tooth displacement, temporomandibular joint–related problems, and postoperative pain, trismus, and edema.^10,12,14–16^

Postoperative pain, orofacial swelling, and trismus are so frequent after third-molar surgery that they are almost regarded as expected sequelae.^17^ These complications chiefly arise from surgical tissue trauma and the ensuing inflammatory response. Pain typically emerges as local anesthesia dissipates, with maximal intensity occurring at approximately 12 hours after the procedure.^18^ Pain severity tends to correlate with operative difficulty and duration, whereas comparable associations have not been consistently demonstrated for the extent of swelling or the degree of mouth-opening limitation (trismus). By contrast, a strong association between postoperative pain and trismus has been documented.^18^ Trismus most commonly results from inflammation of the masticatory musculature, provoking muscle spasm and frequently linked to mucoperiosteal flap detachment;^19,20^ additional etiologies include temporomandibular joint trauma, direct injury to the medial pterygoid muscle during an inferior alveolar nerve trunk block, and, less commonly, infection.^20^ Orofacial edema generally peaks by the end of postoperative day 2 and typically resolves by days 5 to 6.^18,21^ In current clinical practice, nearly 90% of third-molar removals are performed without notable adverse events, although reported rates of major complications vary widely (≈4.6%–30.9%).^22,23^ Identified risk factors include advanced patient age, prior infection, depth of impaction, longer operative time, anatomic variability, tobacco use, oral contraceptive use, and the specific local anesthetic technique used.^12,22–27^

In this context, contemporary strategies increasingly target optimization of the healing cascade itself—particularly through modulation of fibroblast activity.^28^ Polynucleotides (PN) have been shown to increase cell numbers in human fibroblast cultures, enhance protein synthesis, and upregulate collagen types I and III, thereby improving wound healing in vitro and in vivo.^29^ Polynucleotides—gel-based medical devices composed of salified chains of deoxyribonucleic acids—appear to promote collagen deposition while indirectly accelerating tissue recovery without evidence of fibrotic remodeling, addressing concerns that excessive collagen could otherwise predispose to fibrosis and functional impairment.^29,30^ In parallel, high-molecular-weight hyaluronic acid (HA) has emerged as a promising wound-healing adjunct owing to bacteriostatic, anti-inflammatory, and immunomodulatory effects; it participates in key signaling pathways during repair and may facilitate re-epithelialization while enhancing proliferation and migration of human oral fibroblasts and periodontal ligament cells.^31–34^ Preclinical investigations indicate that combining HA with autogenous bone, collagen sponges, or xenografts augments bone formation and reduces residual graft material in surgically created defects;^35–37^ in chronically inflamed extraction sockets, HA accelerated wound closure and improved bone formation, and it expedited margin approximation in excisional palatal wounds.^38,39^ Additional preclinical data support benefits in periodontal regeneration and root coverage, with pro-angiogenic effects and short-term support of bone repair.^40–43^ When used with L-PRF, HA has been associated with superior soft-tissue healing, reduced edema, and decreased analgesic consumption.^44,45^ Clinically, adjunctive HA has yielded improved outcomes in nonsurgical periodontal therapy, regenerative periodontal surgery, and root-coverage procedures, and has shown promise as a filler for papillary reconstruction.^46–58^ Randomized controlled trials further suggest accelerated palatal wound healing and reduced postoperative pain after connective tissue graft harvesting, as well as enhanced healing and comfort following laser-assisted frenectomy.^59,60^ A growing body of evidence indicates that HA promotes wound repair by fostering granulation tissue, tempering excessive inflammation, and supporting re-epithelialization and angiogenesis.^61^

Given the high prevalence of postoperative morbidity after third-molar surgery and the emerging biological rationale for PN and hyaluronic acid, the present split-mouth clinical investigation evaluates the postoperative application of a combined PN–HA-based gel (Regenfast^©^) following the surgical removal of partially impacted mandibular third molars. The primary aim is to assess its effectiveness in attenuating pain, swelling, and trismus and in enhancing early wound healing, relative to conventional management, within the same patient.

## METHODS

### Study Design

This single-center, retrospective, split-mouth comparative study was conducted in accordance with the Declaration of Helsinki (2013 revision). Because the analysis used anonymized data collected during routine care, the institutional ethics committee granted an exemption from formal review. At the time of clinical care, all patients provided written informed consent for the surgical procedures and standard perioperative management.

Eligible cases were consecutively identified from the registry of the Unit of Oral Surgery, University of Catania, between September 2024 and March 2025, selecting healthy adults (American Society of Anesthesiologists Physical Status I) who had undergone surgical removal of both impacted mandibular third molars. In routine practice, one mandibular site received PN–HA gel as an adjunct immediately before suturing, whereas the contralateral site received standard care without adjunctive biomaterial. Thus, each patient contributed a matched pair of sites (test versus control) and served as their own control in a split-mouth fashion. No randomization or allocation concealment was performed, as exposure reflected clinical decision-making at the time of surgery.

To mitigate measurement bias in this retrospective context, outcome assessors for pain, swelling, trismus, and early wound healing analyzed de-identified records and masked 3D files, remaining unaware of group assignment and time point; the operating surgeon, by definition, was not blinded. Data entry and statistical analysis were performed by investigators masked to allocation.

Inclusion criteria were: (1) age 18 to 35 years; (2) good general health with no systemic disease; (3) presence of both impacted mandibular third molars classified as Class II, type B impaction; and (4) no pericoronitis or clinical signs of inflammation within the preceding 30 days.

Exclusion criteria were: (1) regular use of oral contraceptives or other medications; (2) intake of immunosuppressive or anti-inflammatory drugs within 3 months before surgery; (3) pregnancy or lactation; (4) documented excessive alcohol consumption; (5) known allergy to local anesthetics; (6) regular smoking; and (7) any preoperative oral pain score >0 on the Visual Analog Scale (VAS).

Preoperative imaging included panoramic radiography (orthopantomogram) to assess third-molar position; cone-beam CT was obtained when additional 3-dimensional information was required.

### Power and Sample Size

An a priori sample-size calculation was performed for the split-mouth (paired) design, targeting a moderate effect size (Cohen’s d=0.5) with a 2-sided α=0.05 and power (1–β)=0.85. Under these assumptions, 19 participants (ie, 19 matched pairs of surgical sites within patients) were required to detect the planned within-subject difference. Moreover, we successfully enrolled 22 patients, thereby meeting the prespecified recruitment goal and preserving at least the planned 85% power to detect the intended effect size. In plain terms, the study was sized to identify a clinically meaningful difference between the test and control sides within the same individual. Calculations were carried out using a paired-comparison framework appropriate for split-mouth data (G*Power 3.1), ensuring that the within-subject correlation structure was accounted for analytically.

### Preoperative Assessment

After enrollment, all patients received a session of professional oral hygiene. Before surgery, baseline information was recorded, including age, sex, systemic health status, coagulation and glycemic parameters, and periodontal condition. Pre-enrollment panoramic radiographs—and cone-beam CT scans when available—were reviewed to confirm tooth position, depth of impaction, and stage of root development for each impacted mandibular third molar.

As preoperative prophylaxis, patients were prescribed amoxicillin 875 mg with clavulanic acid 125 mg (Augmentin; GlaxoSmithKline) starting 2 days before the scheduled procedure. In addition, a 0.12% chlorhexidine mouth rinse was used twice daily for 3 days before surgery. All interventions were performed by a single experienced oral surgeon (M.C.) following a standardized surgical protocol.

Split-mouth randomization was generated by an independent investigator not involved in patient care or outcome assessment. Each participant contributed 2 surgical sites, which were allocated to:

- Test side (TG): application of a PN–HA gel directly into the postextraction socket immediately after tooth removal and hemostasis, before suturing, according to the manufacturer’s instructions (standardized volume per site).
- Control side (CG): standard care without adjunctive biomaterials (no PN/HA gel).

### Treatment

Local anesthesia was administered by vestibular and lingual infiltrations of 3% mepivacaine with epinephrine 1:100,000, supplemented by an inferior alveolar nerve block using 3% mepivacaine without vasoconstrictor. For each patient, the total anesthetic dose was recorded by counting the number of cartridges used.

A No. 15 scalpel blade was used to make an incision extending from the mesial aspect of the first mandibular molar to the distal aspect of the second mandibular molar, with an additional releasing incision along the ascending ramus when indicated. This approach permitted elevation of a full-thickness mucoperiosteal flap and exposure of the cortical plate.

**FIGURE 1:**
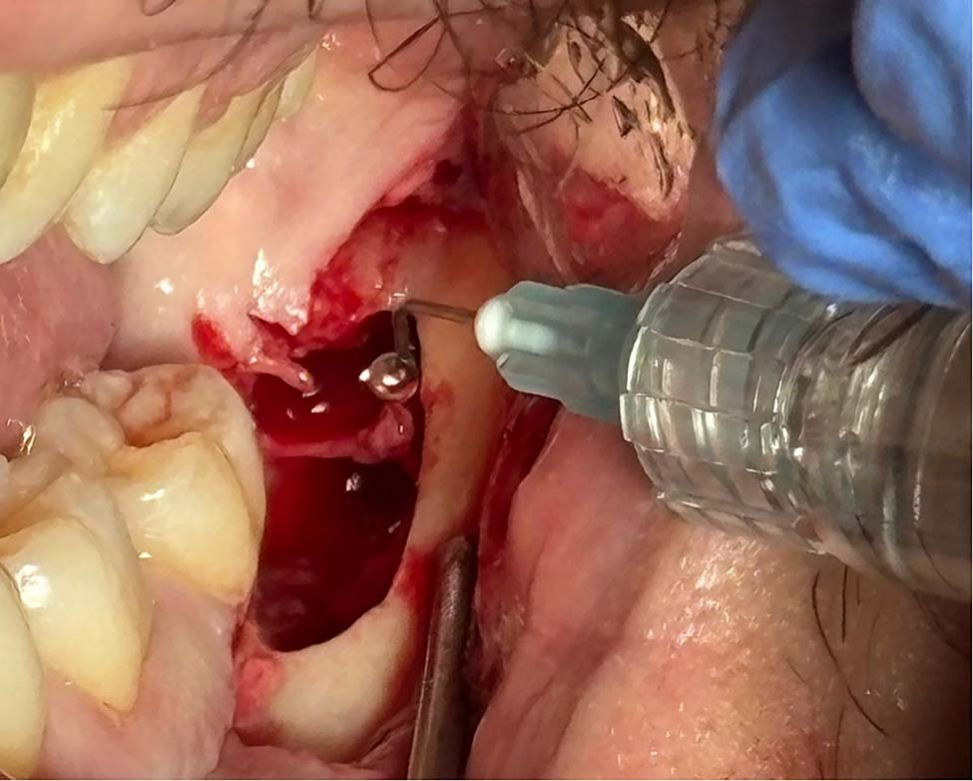
Intrasocket application of a polynucleotide–hyaluronic acid gel (5 mL) to the mandibular third-molar socket immediately before flap closure.

Osteotomy was carried out with a spherical laminated tungsten bur mounted on a surgical handpiece (Ref. 1600383-001, BIEN AIR). When crown or root sectioning was required, a turbine-driven laminated cylindrical tungsten bur was used (Bora L, BIEN AIR; Ref. 1600382-001). After completing the osteotomy and any necessary odontotomy, the tooth was removed. The site was inspected, and in the test group (TG) 5 mL of Regenfast® was applied into the socket before suturing (Fig. 1), while in the control group (CG) no biomaterial was used. Following, the flap was repositioned and sutured with absorbable 3-0 polyglactin 910 sutures (Vicryl 3-0, Ethicon).

All enrolled patients underwent osteotomy and tooth sectioning without intraoperative incidents or complications.

The postoperative regimen included local hemostasis using a compressive gauze for 30 minutes and intermittent cryotherapy—15 minutes each hour during the first 3 hours. Immediately after surgery, patients received detailed written instructions covering postoperative care: adherence to a cold, liquid diet for the first 24 hours; oral hygiene recommendations; and information about expected symptoms (pain, swelling, fever) and potential adverse effects related to the prescribed medications (eg, nausea, vomiting, drug intolerance).

Comprehensive operative records were kept, including the duration of the procedure (from first incision to final suture, in minutes). Patients were instructed to continue antibiotic therapy for an additional 4 days and to take ibuprofen 600 mg every 12 hours for 3 days. Sutures were removed on postoperative day 7. Surgeries on contralateral sides were scheduled 4 weeks apart. Throughout the study, the surgical team remained available to manage any postoperative concerns—such as infection, uncontrolled pain, fever, or other procedure-related complications—and any adverse drug reactions were carefully documented at each follow-up visit.

### Parameters Evaluation

#### Assessment of Postoperative Pain

Pain was assessed on a 0 to 10 Visual Analog Scale (VAS), with 0 indicating no pain and 10 indicating severe pain.^32^ Patients recorded a rating 12 hours after surgery and then once daily, at approximately the same time, for 7 consecutive days.

#### Assessment of Mouth Opening

To assess postoperative trismus, maximum mouth opening was measured preoperatively with a calibrated sliding caliper. The distance was recorded from the incisal edge of tooth 11 to the incisal edge of tooth 41 immediately before surgery.^33^ The same measurement was repeated on postoperative days 2, 5, and 7.

#### Three-Dimensional Assessment of Facial Swelling

Before third-molar extraction, each patient underwent a three-dimensional (3D) facial scan with the ObiScanner (Fifth Ingenium S.r.l.s.), a system developed for digital dentistry that captures a full 3D facial model in approximately 15 seconds. Scans were acquired at 4 time points: (1) baseline before surgery (T0), 2 days postoperatively (T1), 5 days (T2), and 7 days (T3). All scans were exported in .STL format.

To quantify swelling over time, each scan was imported into 3-Matic software (v13.0, Materialise). Following the measurement protocol described by Grossi and colleagues, validated linear surface measurements were obtained using the “measure length” tool. Two linear measurements were recorded among 3 anatomic reference points: (1) the tragus, (2) the soft-tissue pogonion, and (3) the corner of the mouth. For each side, the preoperative values were summed and used as the baseline.

All measurements were exported to a spreadsheet (MS Excel, Microsoft Corporation).

#### Surface Evaluation and Deviation Analysis

For surface-based evaluation, each patient’s postoperative 3D facial model was aligned and superimposed onto the baseline (T0) scan, which served as the reference. Registration proceeded in 2 steps: first, a point-based alignment using 3 stable landmarks (1) left endocanthion (innermost corner of the left palpebral fissure), (2) right endocanthion, and (3) subnasale (midpoint of the columella)—followed by an automated best-fit surface registration to refine the match.

The registered scan pairs (T0 versus each follow-up time point) were then imported into Geomagic Control X (v.2018.1.1, 3D Systems) to compute the percentage of surface correspondence after superimposition. A color-coded deviation map visually represented point-to-point distances: values above the positive threshold appeared from yellow to red, values below the negative threshold from turquoise to dark blue, and distances within tolerance were displayed in green. The tolerance band was set at ±0.5 mm.

### Statistical Analyses

Data were entered and analyzed using SPSS (v25.0; IBM Corp.). For masking, each participant’s records were stored in a coded file linked to a unique study ID, and treatment allocation (TG versus CG) was recorded in sealed code lists. The database labeled observations by group (TG, CG), and decoding of group identifiers was performed only after the clinical phase and the primary statistical analyses were completed. Continuous variables are reported as mean±SD for each treatment condition.

Assumptions of normality and homogeneity of variance were examined with the Shapiro-Wilk and Levene tests, respectively. As these assumptions were met, parametric procedures were applied. Where appropriate, paired *t* tests were used to evaluate within-subject differences at baseline. Changes in the 3 primary outcomes (facial swelling, mouth opening, and pain) across time points were assessed using one-way ANOVA, with Tukey post hoc tests for multiple comparisons. Statistical significance was set at *P*<0.05.

Assuming parametric distributions, linear associations among postoperative outcomes were examined using Pearson’s product–moment correlation coefficient (r). Correlations were computed between pairs of variables (pain versus mouth opening, pain versus swelling, and mouth opening versus swelling) at each postoperative time point, within each allocation (TG and CG), and in the pooled dataset. Two-tailed *P* values and 95% CIs for r were derived using Fisher z transformation. Where multiple correlations were tested simultaneously, familywise error was controlled using a Holm-Bonferroni adjustment. For transparency, scatterplots with least-squares fit lines were inspected to verify linearity and to screen for influential observations.

## RESULTS

**FIGURE 2:**
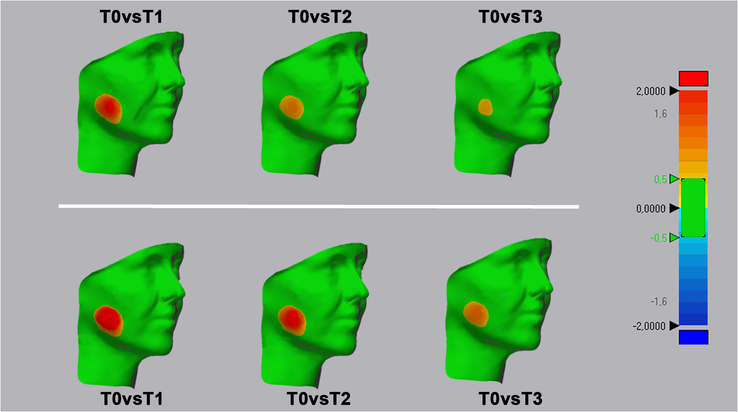
Three-dimensional facial scan acquisition at baseline (T0) versus postoperative time points.

No adverse events were recorded. Specifically, there were no reports of nausea, vomiting, headache, excessive drowsiness or sweating, allergic reactions, or medication intolerance. Soft-tissue healing was uneventful, and no secondary infections occurred. All included cases completed follow-up without postoperative complications. The cohort comprised 18 patients (11 men, 7 women; mean age 26.9±4.0 years), each contributing a test and a control site. Healing was smooth in every case, with no infections or abscesses observed during the observation period. Operative conditions were comparable between sides. Mean procedure time differed by less than 2 minutes (about 27 minutes on the test side versus 25 minutes on the control), a difference that did not reach statistical significance. Pain peaked around 12 hours after surgery on both sides and then declined steadily over the first week. Throughout the early window (12–72 h), test sites treated with the PN–HA gel reported consistently lower VAS scores than control sites, most clearly at 12 hours and on days 2 to 3 (group differences typically around 0.7–0.9 points; *P*<0.05). By day 5, scores had converged, and by day 7 pain was minimal in both allocations. In line with these trends, patients took slightly fewer ibuprofen tablets for the test side during the first 72 hours. Three-dimensional facial assessments mirrored the pain findings. Swelling was greatest at day 2 and then resolved toward baseline by day 7. Test sides showed smaller early increases in linear facial measurements relative to baseline—most evident at day 2 and still appreciable at day 5—while by day 7 differences were negligible. Color-mapped deviation analyses qualitatively confirmed reduced outward displacement over the masseteric–buccal region on the test side (Fig. 2). Mouth opening followed the expected postoperative course. Maximum interincisal distance dropped after surgery, with a milder limitation on the test side at days 2 and 5; by day 7, mouth opening had largely recovered in both allocations, with only a small, nonsignificant advantage for the test side (Table 1, Supplemental Digital Content 1, http://links.lww.com/SCS/I881).

Correlation analyses supported known relationships among outcomes. In the pooled dataset across all time points, pain versus mouth opening showed a moderate inverse correlation (Pearson’s r=−0.56, 95% CI: −0.68 to −0.41; *P*<0.001), pain versus swelling a moderate positive correlation (r=0.42, 95% CI: 0.25 to 0.56; *P*<0.001), and swelling versus mouth opening a weaker inverse correlation (r=−0.31, 95% CI: −0.47 to −0.13; *P*=0.002). Effects were strongest at the inflammatory peak: day 2—pain versus mouth opening r=−0.62; pain versus swelling r=0.48; swelling versus mouth opening r=−0.36 (all *P*≤0.01). Day 3 values were similar (−0.58, 0.44, −0.33; all *P*≤0.01). Within allocations, patterns were broadly comparable, with slightly larger magnitudes in the control sites at day 2 (eg, pain versus mouth opening r=−0.65 in CG versus −0.58 in TG) (Table 2, Supplemental Digital Content 1, http://links.lww.com/SCS/I881).

## DISCUSSION

Surgical extraction of mandibular third molars commonly triggers an acute inflammatory response—pain, swelling, and transient limitation of mouth opening—that can impair patient well-being in the first postoperative days. In this retrospective split-mouth comparative study, we investigated whether adjunctive placement of a PN–HA gel would mitigate these early sequelae relative to standard care without biomaterials. All cases completed follow-up without complications, and healing was uniformly uneventful, supporting the safety of the intervention in routine practice. Importantly, operative time was comparable between allocations (approx. 27 versus 25 minutes for test and control sites, respectively), indicating that gel application does not prolong surgery. Our findings indicate a modest but clinically coherent early benefit of the PN–HA adjunct. Pain, which peaked at ∼12 hours on both sides, was lower on test sites during the first 48 to 72 hours (eg, 12 h: 5.2±1.3 versus 6.1±1.4; day 2: 4.1±1.2 versus 4.9±1.3), with between-side differences tapering thereafter and converging by day 5 to 7. Consistent with this pattern, early analgesic consumption was slightly reduced for test sites over the first 72 hours. Three-dimensional facial assessment corroborated the analgesic signal: swelling was greatest at day 2 and diminished toward baseline by day 7, with smaller early increases on the test side (day 2: +2.6±0.9 mm versus +3.4±1.0 mm; day 5: +1.2±0.7 mm versus +1.8±0.8 mm). Functional recovery echoed these trends: the reduction in maximum interincisal distance was milder on test sites at days 2 and 5 (eg, day 2: 33.5±4.1 mm versus 31.2±4.4 mm), with near-complete recovery on both sides by day 7. Together, these data suggest that PN–HA primarily benefits the early inflammatory window without altering the overall trajectory of recovery. The correlation analyses help contextualize these outcomes and align with established postoperative dynamics. Across all time points, higher pain scores correlated with more limited mouth opening (r=−0.56; 95% CI: −0.68 to −0.41) and, to a lesser extent, with greater swelling (r=0.42; 95% CI: 0.25–0.56), while swelling and mouth opening showed a weaker inverse relationship (r=−0.31; 95% CI: −0.47 to −0.13). These associations strengthened around the inflammatory peak (day 2: −0.62, 0.48, and −0.36, respectively), suggesting that the symptomatic triad is most tightly coupled when tissue edema and nociception are maximal. Patterns were broadly similar in both allocations, with slightly stronger correlations on control sites during days 2 to 3—consistent with the hypothesis that PN–HA dampens the inflammatory cascade, thereby loosening the coupling between symptoms.

Mechanistically, these clinical effects are plausible. Hyaluronic acid can modulate early inflammation, support granulation tissue, and facilitate re-epithelialization, while PN enhances fibroblast activity and collagen I/III expression without promoting pathologic fibrosis. The observed reduction in early pain and edema, along with faster functional rebound, fits this biological rationale. Notably, our 3D facial scanning protocol provided reproducible, operator-independent quantification of swelling and surface deviations—an advantage over traditional linear measurements alone—and the qualitative deviation maps mirrored the numeric improvements seen on the test side.

This study’s strengths include the split-mouth design (controlling for inter-individual variability), standardized surgical workflows, blinded assessment of swelling and mouth opening using de-identified files, and triangulation of outcomes (symptoms, function, and 3D morphology). Nonetheless, several limitations should be acknowledged. The retrospective design introduces inherent selection and information biases despite efforts to mask outcome assessment. The single-center setting and modest sample size limit generalizability and preclude definitive subgroup analyses. Finally, while early benefits are evident, our follow-up horizon focuses on the first postoperative week; longer observation would clarify whether any differences persist beyond day 7.

Studies in third-molar surgery indicate that intrasocket HA reduces early postoperative morbidity—principally pain and edema—with trajectories returning toward baseline by approximately day 5 to 7.^61–63^ Alenazi et al^61^ reported improved wound healing and lower discomfort after impacted third-molar removal with intrasocket HA, mirroring the typical peak-and-decline profile of edema within 48 hours and attenuation by 1 week.^61^ Shuborna et al likewise observed reductions in pain, swelling, and trismus when HA was placed into extraction sockets compared with control treatment.^62^ These clinical signals are reinforced by newer RCT data showing 0.8% HA gel can advance soft-tissue healing and early bone density within extraction sites.^63^

The biochemical rationale for these effects is well described. Hyaluronic acid is a ubiquitous extracellular-matrix glycosaminoglycan that provides a highly hydrated, viscoelastic scaffold supporting cell migration and proliferation; it also engages hyaladherins at the cell surface to modulate signaling.^64–67^ During repair, HA can dampen excessive inflammation, promote granulation tissue, facilitate angiogenesis, and accelerate re-epithelialization—mechanisms that plausibly underlie early reductions in pain and swelling and smoother soft-tissue recovery after oral surgery.^64–67^ These actions have been documented across model systems and reviews spanning skin and oral tissues, including molecular-weight–dependent effects on keratinocytes and fibroblasts (eg, low- to intermediate-MW HA enhancing epithelial closure and coordinated inflammatory resolution).^64,66,67^

At the same time, formulation and delivery matter. Studies using injectable HA at higher concentrations have noted hemostatic perturbations (eg, prolonged bleeding time) and less favorable edema profiles, suggesting that concentration, route, and perioperative protocols can shape outcomes—an important consideration when comparing across trials and when favoring intrasocket gel placement for early symptomatic relief.^68^

Beyond third-molar surgery, periodontal meta-analyses show adjunctive HA can yield additional clinical benefits (eg, improved probing depths and clinical attachment) when added to nonsurgical periodontal therapy, supporting a broader anti-inflammatory and pro-healing role of HA within oral tissues. These external data strengthen the biological and clinical plausibility of early postoperative advantages observed when HA is applied at the socket level.^69–71^

Building on the HA comparator framework, adding PN to HA provides a biologically coherent route to amplify early soft-tissue healing. In primary human gingival fibroblasts, a PN–HA hydrogel increased cell number and viability, total protein content, and clonogenic efficiency, and promoted dense, multilayered colony formation. PN–HA also upregulated COL1A1/COL3A1 expression and accelerated wound closure in scratch assays—achieving complete closure by 48 hours when comparison groups had not bridged the defect by 96 hours.^72^ These data support a model in which PN–HA augments fibroblast activity, extracellular-matrix deposition, and coordinated migration—programs that plausibly mitigate early pain and edema clinically by stabilizing the wound bed and hastening mucosal coverage.^72^

The biophysical contribution of PN complements HA’s hydrated scaffold: PN polymers form a viscoelastic gel and are progressively cleaved to oligonucleotides that can sustain cellular proliferation during repair (eg, increased colony density and rapid monolayer repopulation), while HA facilitates organized stratification and matrix architecture—features consistent with an early clinical benefit (first 48–72 h) without evidence of aberrant fibrosis.^72^

In an in vivo rabbit maxillary sinus augmentation model, mixing PN–HA gel with deproteinized bovine bone mineral did not increase new bone formation at 2 or 10 weeks compared with xenograft alone (new bone ≈8% at 2 weeks and ≈27%–28% at 10 weeks in both groups). Mucosal thinning and occasional perforations related to sharp granule contours were similar between groups.^73^ These results suggest PN–HA does not necessarily potentiate osteogenesis when combined with a slowly resorbing xenograft in a nonloaded sinus environment—an outcome distinct from soft-tissue endpoints and consistent with PN–HA primarily benefiting fibroblast-mediated mucosal repair rather than bone accrual in this setting.^73^

Taken together, current evidence indicates that PN–HA is most likely to deliver clinical benefit where fibroblast dynamics and epithelial coverage dominate the healing trajectory—as in third-molar sockets—by accelerating early soft-tissue closure and stabilizing the inflammatory milieu. Conversely, hard-tissue regeneration appears more dependent on the graft’s osteoconductive properties, host bone biology, and mechanical environment than on fibroblast-centric adjuncts, helping reconcile early improvements in pain, swelling, and mouth opening with laboratory findings.^72,73^

### Limitations and Future Prospectives

The modest sample size limits precision and generalizability. A larger cohort would enable adequately powered subgroup analyses (impaction depth/angulation, flap design, operative time, smoking status) and yield more robust effect estimates. Future work should also ensure balanced representation across age and sex strata and across Winter/impaction classes, and incorporate predefined responder thresholds.

## CONCLUSIONS

PN–HA gel improves early postoperative recovery after removal of partially impacted mandibular third molars: lower pain and reduced facial swelling in the first 48 to 72 hours, a quicker rebound in mouth opening, and no increase in operative time or adverse events. Correlation patterns were as expected—higher pain tracked with greater swelling and reduced mouth opening, peaking around the inflammatory window—suggesting that PN–HA attenuates early inflammatory responses. These outcomes converge with the literature on intrasocket HA and point to a complementary, fibroblast-driven contribution from PN to soft-tissue repair. Strengths include a split-mouth framework, standardized surgery, and blinded 3D assessments; limitations include retrospective design, single center, modest sample size, and 1-week follow-up. Within these bounds, PN–HA emerges as a safe, practical adjunct to reduce short-term morbidity after third-molar surgery.

## Supplementary Material
