# Supplementary material for: Effect of a Polynucleotide-Hyaluronic Acid Gel on Mandibular Third-Molar Extraction Discomfort
Source: J Craniofac Surg. 2025 Dec 31;37(6):1693–9. doi: 10.1097/SCS.0000000000012307 (PMC13200887; doi:10.1097/SCS.0000000000012307)
Supplement: Supplementary file 1 [file scs-37-1693-s001.docx]

## Table 1. Cohort, Safety, and Operative Characteristics

| VARIABLE | VALUE |
| --- | --- |
| Patients, n (men/women) | 18 (11/7) |
| Age, years (mean ± SD) | 26.9 ± 4.0 |
| Follow-up completion | 100% |
| Adverse events | None recorded |
| Specific AEs (nausea, vomiting, headache, excessive drowsiness/sweating, allergy, drug intolerance) | 0 for each |
| Soft-tissue healing | Uneventful in all cases |
| Secondary infections/abscesses | 0 |
| Operative time, min (mean ± SD) — TG | 27.1 ± 4.5 |
| Operative time, min (mean ± SD) — CG | 25.3 ± 4.0 |

## Table 2. Efficacy Outcomes (Test vs Control) and Correlations

| OUTCOME | TIME POINT | TEST (PN–HA) | CONTROL (NO ADJUNCT) | P VALUE / NOTE |
| --- | --- | --- | --- | --- |
| Pain (VAS 0–10) | 12 h | 5.2 ± 1.3 | 6.1 ± 1.4 | 0.01 |
|  | Day 2 | 4.1 ± 1.2 | 4.9 ± 1.3 | 0.02 |
|  | Day 5 | — | — | Groups converged by Day 5–7 |
|  | Day 7 | Minimal in both | Minimal in both | — |
| Facial swelling (Δ mm vs baseline) | Day 2 | +2.6 ± 0.9 | +3.4 ± 1.0 | 0.01 |
|  | Day 5 | +1.2 ± 0.7 | +1.8 ± 0.8 | 0.02 |
|  | Day 7 | ~0.3–0.5 | ~0.5 | Differences negligible |
| Maximum interincisal distance (mm) | Day 2 | 33.5 ± 4.1 | 31.2 ± 4.4 | 0.04 |
|  | Day 5 | 39.8 ± 3.7 | 37.5 ± 3.9 | 0.03 |
|  | Day 7 | Largely recovered | Largely recovered | Small, non-significant advantage |
| Deviation map | Day 2–5 | Smaller outward displacement over masseteric–buccal region | Greater displacement | Visual confirmation of swelling data |
| Correlation coefficients (Pearson r) | All time points (pooled) |  |  |  |
| Pain vs mouth opening | — | r = −0.56 (95% CI −0.68 to −0.41) |  | P < 0.001 |
| Pain vs swelling | — | r = 0.42 (95% CI 0.25 to 0.56) |  | P < 0.001 |
| Swelling vs mouth opening | — | r = −0.31 (95% CI −0.47 to −0.13) |  | P = 0.002 |
| Correlation coefficients (peak window) | Day 2 |  |  |  |
| Pain vs mouth opening |  | r = −0.62 |  | P ≤ 0.01 |
| Pain vs swelling |  | r = 0.48 |  | P ≤ 0.01 |
| Swelling vs mouth opening |  | r = −0.36 |  | P ≤ 0.01 |
| Correlation coefficients (peak window) | Day 3 |  |  |  |
| Pain vs mouth opening |  | r = −0.58 |  | P ≤ 0.01 |
| Pain vs swelling |  | r = 0.44 |  | P ≤ 0.01 |
| Swelling vs mouth opening |  | r = −0.33 |  | P ≤ 0.01 |
| Allocation comparison at Day 2 | Pain vs mouth opening | CG: r = −0.65; TG: r = −0.58 |  | Slightly stronger in CG |
